# Foliar application of pyroligneous acid acts synergistically with fertilizer to improve the productivity and phytochemical properties of greenhouse-grown tomato

**DOI:** 10.1038/s41598-024-52026-2

**Published:** 2024-01-22

**Authors:** Raphael Ofoe, Seyed Mohammad Nasir Mousavi, Raymond H. Thomas, Lord Abbey

**Affiliations:** 1 Department of Plant, Food, and Environmental Sciences, Faculty of Agriculture, Dalhousie University, 50 Pictou Road, Bible Hill, NS B2N 5E3 Canada; 2 Department of Biology, Faculty of Science, Western University 2025E Biological and Geological Sciences Building, 1151 Richmond Street, London, ON N6A 5B7 Canada

**Keywords:** Plant development, Plant physiology

## Abstract

Pyroligneous acid (PA) is rich in bioactive compounds and known to have the potential to improve crop productivity and phytochemical content. However, the synergistic effect of PA and fertilizer has not been thoroughly studied. In this study, we assessed the biostimulatory effect of different rates of foliar PA application (i.e., 0, 0.25, 0.5, 1, and 2% PA/ddH_2_O (*v/v*)) combined with full rate (i.e., 0.63, 0.28, 1.03 g) and half rate of nitrogen-phosphorus-potassium (NPK) fertilizer on the yield and nutritional quality of greenhouse-grown tomato (*Solanum lycopersicum* ‘Scotia’). Plants treated with 0.25% and 0.5% PA showed a significantly (*p* < 0.001) higher maximum quantum efficiency of photosystem II (*Fv/Fm*) and increased potential photosynthetic capacity (*Fv/Fo*), especially when combined with the full NPK rate. Leaf chlorophyll was significantly (*p* < 0.001) increased by approximately 0.60 and 0.49 folds in plants treated with 2% PA and full NPK rate compared to no spray and water, respectively. Total number of fruits was significantly (*p* < 0.001) increased by approximately 0.56 folds with the 2% PA irrespective of the NPK rate. The combined 2% PA and full NPK rate enhanced total fruit weight and the number of marketable fruits. Similarly, fruit protein, sugar and 2,2-diphenyl-1-picrylhydrazyl (DPPH) activity were significantly (*p* < 0.001) enhanced by the combined 2% PA and full NPK rate. In contrast, the 0.5% PA combined with half NPK rate increased fruit carotenoid and phenolic contents while the 2% PA plus half NPK rate enhanced fruit flavonoid content. Generally, the synergistic effect of PA and NPK fertilizer increased fruit elemental composition. These showed that foliar application of 2% PA with full NPK rate is the best treatment combination that can be adopted as a novel strategy to increase the productivity and quality of tomato fruits. However, further study is required to investigate the molecular basis of PA biostimulatory effect on plants.

## Introduction

Tomato (*Solanum lycopersicum*) is the most cultivated greenhouse and consumed vegetable worldwide due to its diverse use in sauces, soups and puree^1^. Its fruits are known as a potent source of health-enhancing phytochemicals including phenolics, flavonoids, polysaccharides, vitamins and carotenoids, which makes it a good model for studying fruit quality^2^. These compounds are known to mitigate the destructive effects of reactive oxygen radicals produced during cellular oxidative stress. Dietary intake of tomato fruits has been reported to stimulate antioxidant effects and help prevent chronic diseases in humans including cancers, atherosclerosis, cardiovascular, neurodegenerative and inflammation disorders^2,3^.

Moreover, tomato productivity and phytochemical properties are highly dependent on growing medium fertility status, growing conditions and plant genetic characteristics^4^. It has been reported that appropriate fertilization enhances soil fertility and promotes crop yield^4^. Additionally, the high demand for horticultural crops has resulted in an extensive use of chemical fertilizers for crop production^5^. Intensification of crop production with chemical fertilizer has resulted in increased yield of several crops including wheat (*Triticum aestivum*), maize (*Zea mays*) and tomato^4,5^. However, the indiscriminate use of chemical fertilizers coupled with their negative impact on the environment has become a major concern not only to growers but also to consumers and the public^6^. Evidence suggests that synthetic chemical fertilizers can affect fruit quality including nutritional and phytochemical potentials^4,7,8^. Consequently, the horticultural industry is seeking alternative strategies to boost crop productivity without compromising nutritional qualities while ensuring environmental sustainability^9^.

One of the promising inputs to tackle the concerns of indiscriminate use of chemical fertilizers is the use of pyroligneous acid (PA), which is also known as wood distillate, wood vinegar or liquid smoke^10^. PA is an aqueous translucent reddish-brown liquid that is produced during the pyrolysis of organic biomass^10^. As a natural byproduct of pyrolysis, it contains 80–90% water and is rich in over 200 water-soluble bioactive compounds including organic acids, esters, polyphenols, alcohols, sugar derivatives and mineral elements^10–12^. However, these chemical properties are dependent on the feedstock, heating rate, temperature and resident time^10,11^. PA has been used extensively in agriculture as a biostimulant to increase seed germination and seedling growth^13–15^, crop photosynthetic performance^16–19^, crop biomass, crop yield (fruit number and weight)^20–23^ and the nutrition and phytochemical properties of food crops^17,24–26^. Additionally, PA has recently been used as an antimicrobial agent to control major plant diseases^27,28^. Intriguingly, several studies have reported that PA contains a thermal-resistant biologically active compound known as karrikins, which can remain efficacious at a broad array of concentrations^29,30^. The mode of action of karrikins in plants has been suggested to resemble that of known phytohormones and demonstrated to stimulate seed germination and plant growth^29–32^. Also, the high phenolic content of PA has been proven to exhibit high scavenging activities of reactive oxygen species (ROS) radicals, anti-lipid peroxidation and reducing power activities^11,12,33^. This suggests that a suitable PA rate and concentration can enhance plant growth and productivity.

According to Drobek, et al.^34^, crop production utilizes a huge proportion of fertilizer nutrients with a significant amount not taken up by plants. As such, biostimulants including PA have become a feasible alternative to promote efficient nutrient uptake for increasing crop growth and productivity. Like other biostimulants, PA cannot be defined as a fertilizer since it might not supply direct nutrients to plants but can be treated as an additive fertilizer which facilitates nutrient uptake via stimulation of metabolic and biochemical processes in plants and soils^34,35^. It was demonstrated that co-application of PA with other soil amendments including manure^35,36^, biochar^37^ and compost^35,38^ synergistically improves growth and productivity of crops. Nevertheless, studies on biostimulatory effect of foliar PA application in combination with varying fertilizer regimes on greenhouse tomato yield and fruit quality are limited^20,24^. Besides, the complex composition of PA with its high acidity makes the concentration for use in crop production critical. Therefore, a low PA concentration can contain the right amounts of bioactive compounds which can maximise its effectiveness on crops.

In the present study, we investigated the biostimulatory effect of PA at varying concentrations and in combination with different application rates of NPK fertilizer on the yield and nutritional quality of greenhouse-grown tomato ‘Scotia’. Additionally, the phytochemical levels of leaf tissues can be influenced by mineral nutrition and have been demonstrated to increase following PA application in the leaves of several crops including lettuce^39^, strawberry^25^ and tobacco^19^. As such, we examined the phytochemical contents in treated leaf tissues in comparison to fruit tissue as an alternative strategy for enhancing bioactive compounds in plants.

## Results

### Plant physiology and biomass response

**Figure 1:**
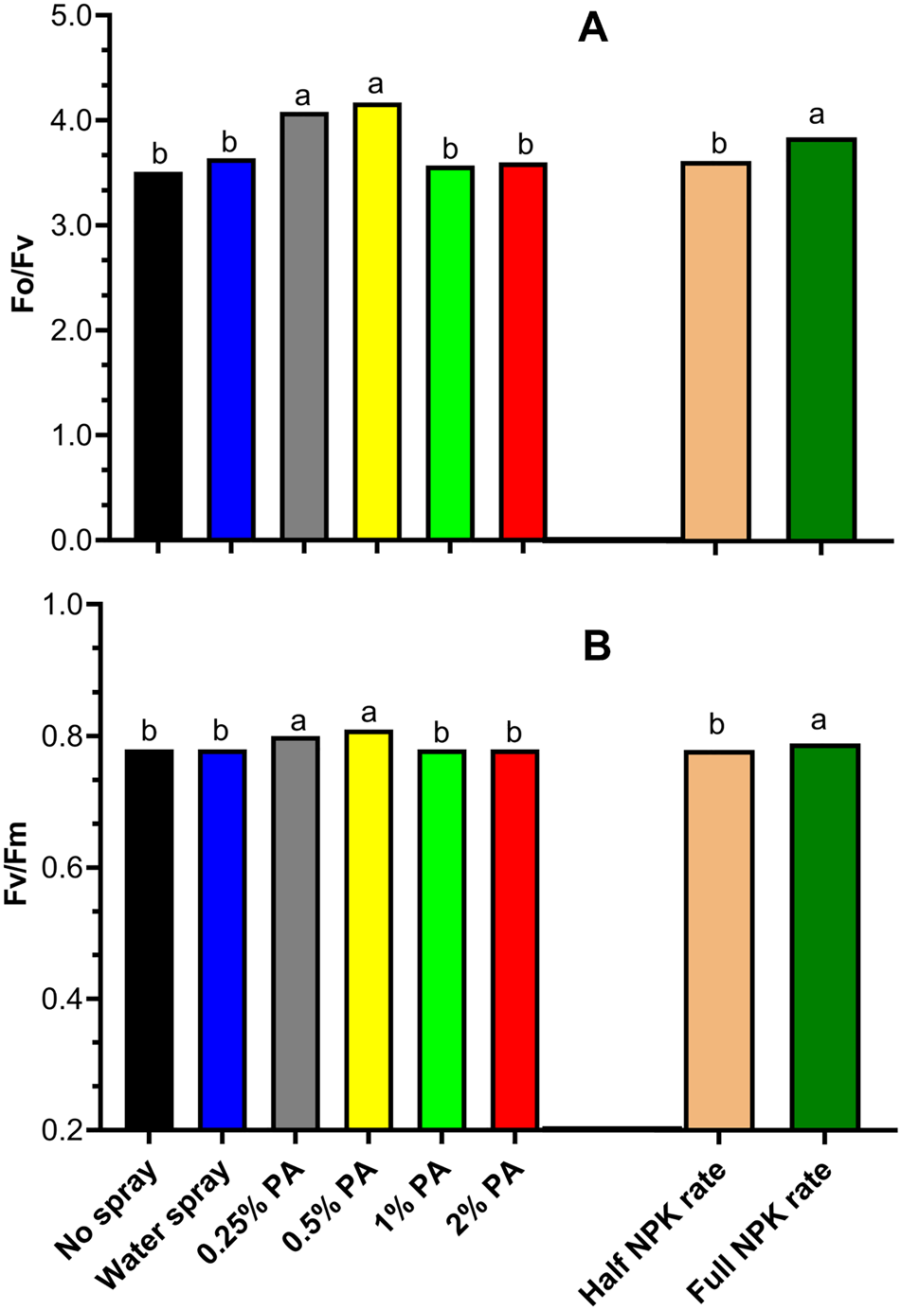
Chlorophyll fluorescence of tomato ‘Scotia’ in response to pyroligneous acid treatment under varying NPK rates. (**A**) Potential photosynthetic capacity (Fv/Fo) (**B**) Maximum quantum efficiency (Fv/Fm). The different letters indicate significant (*p* < 0.05) difference according to Fisher’s least significant difference (LSD) post hoc test.

Chlorophyll fluorescence indices were significantly (*p* < 0.001) affected by PA treatment but not its interaction with NPK (Fig. 1). The results showed that *F*_*v*_*/F*_*o*_ was significantly (*p* < 0.001) increased by *ca.* 0.12 and 0.15 folds in the 0.25% and 0.5% PA-treated plants respectively, compared to no spray plants (Fig. 1A). Similarly, *F*_*v*_*/F*_*m*_ was substantially (*p* < 0.001) enhanced in the 0.25% and 0.5% PA-treated plants compared to no spray plants (Fig. 1B). However, the values of these parameters were reduced as PA was increased to 1% and 2% but was not significantly (*p* > 0.05) different from those of the no spray and water alone treatments (Fig. 1). Analysis of leaf chlorophyll content indicates that PA application had a significant (*p* < 0.001) interaction with NPK (Table 1). The 2% PA with full NPK rate increased total chlorophyll content by *ca*. 0.60 and 0.49 folds compared to the no spray and water alone treatments (Table 1). Moreover, 0.5% PA with full NPK rate significantly (*p* < 0.001) increased only chlorophyll a by *ca.* 0.39 and 0.32 folds compared to no spray and water treatments. Also, the 2% PA treatment with full NPK rate considerably enhanced both chlorophyll a and b contents by *ca*. 0.43 and 0.36 folds and 1.04 and 0.83 folds compared to no spray and water treatments respectively. On the contrary, 0.25% PA in addition to the full NPK rate recorded the least leaf chlorophyll content and was not significantly (*p* > 0.05) different from no spray treatment (Table 1). It was clear that PA slightly increased chlorophylls a and b although they were reduced in plants applied with half NPK rate. Total above-ground fresh weight was significantly (*p* < 0.05) increased by the interaction of PA and NPK but not PA alone (Table 1, Table S1). Overall, the application of PA significantly (*p* < 0.05) increased the above-ground fresh weight under full NPK rate in the range 0.31 and 0.12 folds compared to no spray but not significantly (*p* > 0.05) different from the water-sprayed plants (Table 1). Furthermore, the interaction between PA and NPK had no significant (*p* > 0.05) effect on the above-ground dry weight. However, the above-ground dry weight of 0.25% PA-treated plants under full NPK rate increased by *ca.* 0.54 and 0.11 folds compared to no spray and water treatment, respectively.

**Table 1:** Morpho-physiological response of tomato ‘Scotia’ plant sprayed with pyroligneous acid (PA) under varying NPK rates.

PA	NPK rate	Chlorophyll a (mg g^−1^ FW)	Chlorophyll b (mg g^−1^ FW)	Total chlorophyll (mg g^−1^ FW)	Above-ground fresh weight (g)
0.25%	Full	191.36 e	83.81 g	274.76 f.	562.52 a
	Half	241.14 c	118.28 c	359.02 c	449.41 bcd
0.5%	Full	278.48 a	152.11 b	419.23 b	542.29 ab
	Half	207.04 de	96.64 def	303.35 def	411.41 cd
1%	Full	253.72 b	122.32 c	375.70 c	558.47 a
	Half	191.73 e	86.31f g	277.47 f.	378.59 d
2%	Full	286.20 a	178.83 a	458.29 a	480.85 abc
	Half	214.47 d	100.93 d	302.22 def	471.22 abc
No spray	Full	200.00 de	87.75 efg	287.21 ef	428.57 cd
	Half	215.24 d	99.25 d	313.90 de	426.94 cd
Water	Full	210.93 d	97.69 de	308.14 def	551.14 ab
	Half	233.58 c	101.51 d	327.02 d	413.31 cd

Sources of Variation		*p*-value			
PA		0.001	< 0.001	< 0.001	0.313
NPK		< 0.001	< 0.001	< 0.001	< 0.001
PA × NKP		< 0.001	< 0.001	< 0.001	0.044

The different letters indicate significant (*p* < 0.05) difference according to Fisher’s least significant difference (LSD) post hoc test.

### Fruit yield and marketability

**Figure 2:**
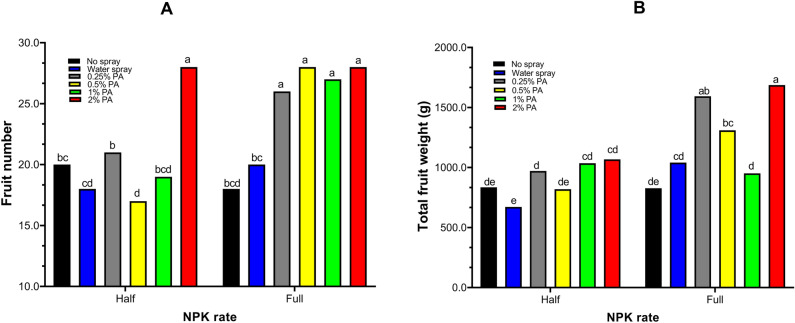
Fruit yield of tomato ‘Scotia’ in response to pyroligneous acid treatment under varying NPK rates. (**A**) Fruit number (**B**) Total fruit weight. The different letters indicate significant (*p* < 0.05) difference according to Fisher’s least significant difference (LSD) post hoc test.

The total number of fruits per plant was significantly (*p* < 0.001) affected by PA and NPK interaction (Fig. 2A, Table S1). The application of both PA and full NPK rate had the most significant (*p* < 0.001) increase in the total number of fruits compared to water and no spray treatments. Notably, PA increased the number of fruits under full NPK rate by *ca.* 0.44–0.56 folds and 0.30–0.40 folds compared to no spray and water alone treatment, respectively. Similarly, the number of fruits was significantly (*p* < 0.001) increased with 2% PA under half NPK rate by *ca.* 0.40 and 0.56 folds compared to no spray and water treatment, respectively (Fig. 2A). However, 0.5% PA and 1% PA slightly reduced the number of fruits although not significantly (*p* > 0.05) different from the no spray and water treatments (Fig. 2A). Moreover, total fruit weight was highest in 2% PA-treated plants under full NPK rate although not significantly (*p* > 0.05) different from 0.25% PA-treated plants (Fig. 2B). The 2% PA significantly (*p* < 0.001) increased total fruit weight by *ca.* 1.04 and 0.62 folds, while the 0.25% PA substantially (*p* < 0.001) increased the total fruit weight by *ca.* 0.93 and 0.53 folds compared to no spray and water treatment respectively (Fig. 2B). Also, the total fruit weight of 0.5% PA-treated plants under full NPK rate was slightly higher than those of the no-spray and water-treated plants but was not significantly (*p* > 0.05) different from that of water-treated plants (Fig. 2B). Nevertheless, under half NPK rate, the total fruit weight of PA-treated plants was significantly (*p* < 0.001) reduced compared to their full NPK rate counterpart.

**Figure 3:**
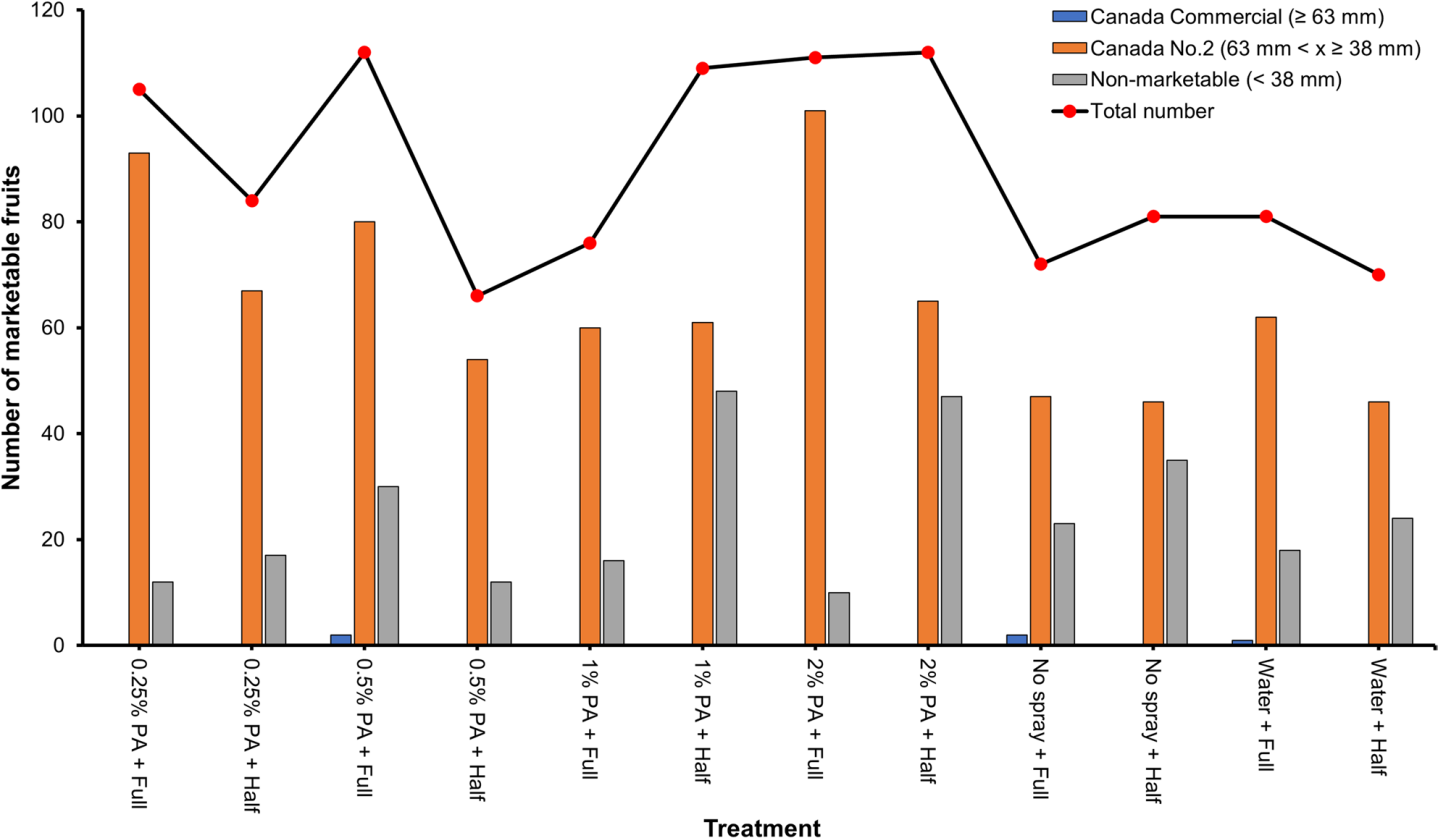
Fruit marketability of tomato ‘Scotia’ plants treated with pyroligneous acid (PA) under varying NPK rates.

To examine the effect of PA on the marketability of tomato fruits, the harvested fruits were graded according to the CFIA grade compendium for fresh vegetables^40^. The results revealed that majority of the fruits (*ca*. 73%) were categorised under Canada No.2 with an equatorial diameter greater than or equal to 38 mm but less than 63 mm, while 27% and 0.2% constituted non-marketable fruits and Canada commercial with an equatorial diameter of less than 38 mm and greater than or equal to 63 mm respectively (Fig. 3, Table S2). Among the various treatment, the 2% PA with full NPK rate recorded the highest increase in the number of Canada No.2 marketable fruits by *ca.* 1.15 and 0.63 folds while non-marketable fruit was reduced by *ca.* 0.57 and 0.44 folds compared to no spray and water treatments, respectively (Fig. 3, Table S2). A similar increase in the number of Canada No.2 marketable fruits was noted with 0.25% and 0.5% PA in combination with the full NPK rate. Nonetheless, the number of Canada No.2 marketable fruits were slightly reduced by 0.25%, 0.5% and 2% PA in combination with half NPK rate compared to full NPK (Fig. 3). Besides, the number of Canada No.2 marketable fruits from 0.25%, 0.5% and 2% PA-treated plants under half NPK rate were comparable to those of no spray and water alone treatments. The number of non-marketable fruits was lower in 0.25% and 0.5% PA-treated plants under half NPK rate but increased in the 1% and 2% PA-treated plants compared to that of no spray and water-treated plants (Fig. 3).

### Fruit chemical composition

**Figure 4:**
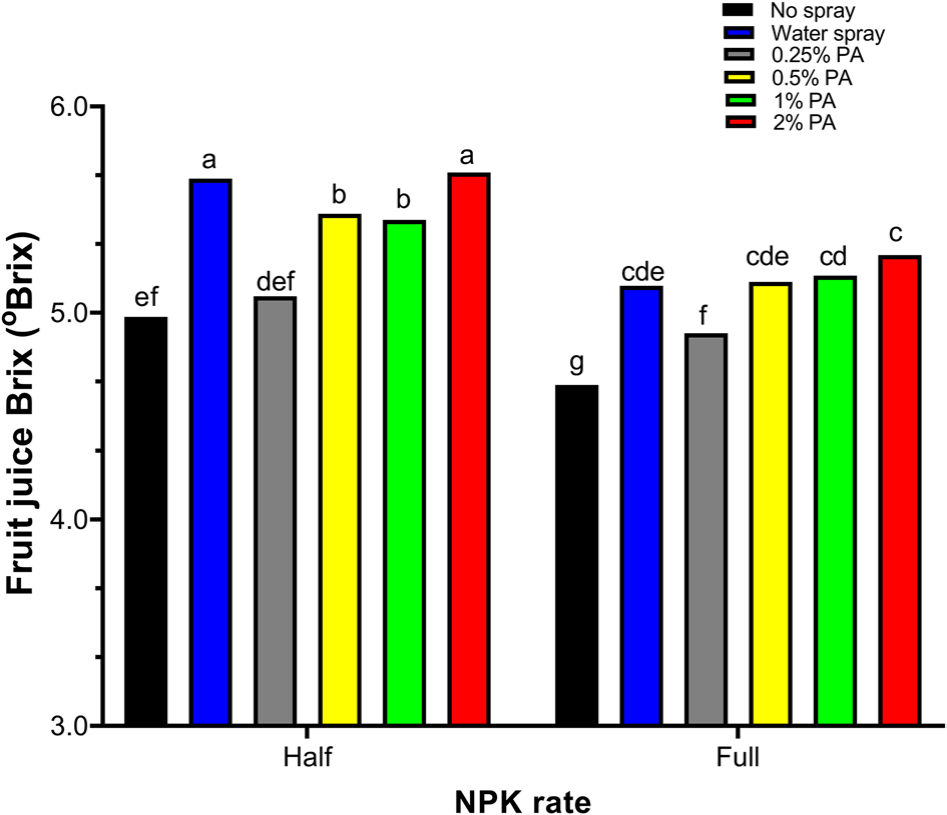
Fruit Brix content of tomato ‘Scotia’ plants treated with pyroligneous acid (PA) under varying NPK rates. The different letters indicate significant (*p* < 0.05) difference according to Fisher’s least significant difference (LSD) post hoc test.

**Figure 5:**
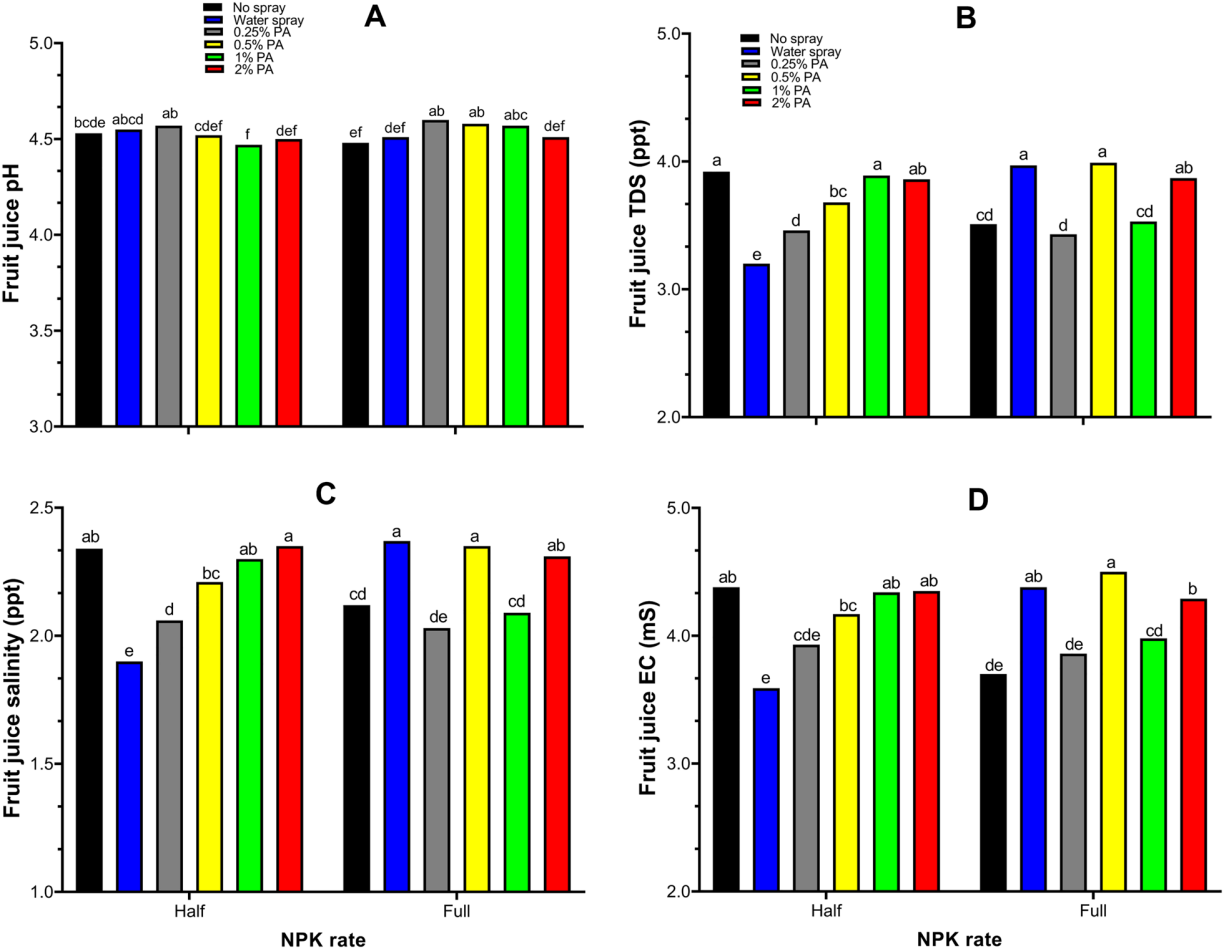
Chemical quality of tomato ‘Scotia’ fruits from plants treated with pyroligneous acid (PA) under varying NPK rates. The different letters indicate significant (*p* < 0.05) difference according to Fisher’s least significant difference (LSD) post hoc test.

Fruit chemical quality parameters including juice Brix, pH, TDS, salinity and EC were significantly (*p* < 0.001) affected by PA and NPK fertilizer interaction (Fig. 4, Fig. 5). Juice Brix content was significantly (*p* < 0.001) increased by *ca.* 0.14 folds following 2% PA with half NPK rate application compared to no spray treatment (Fig. 4). Fruit Brix content of the 2% PA-treated plants was comparable to that of the water-treated plants under half NPK rate. However, the 0.25% PA combined with half NPK rate significantly (*p* < 0.001) reduced Brix content compared to the water treatment but was not different from no spray-treated plants (Fig. 4). Comparatively, Brix content was not significantly (*p* > 0.05) altered in PA-treated plants with full NPK rate except for the 0.25% PA which reduced the Brix content compared to water-treated plants. On the other hand, all PA treatments (except 0.25% PA) with full NPK rate significantly (*p* < 0.001) increased Brix content compared to no spray-treated plants (Fig. 4).

Similarly, 0.25% PA with half NPK rate enhanced fruit juice pH although not significantly (*p* > 0.05) different from the no spray and water treatment (Fig. 5A). Under full NPK rate, all PA treatments significantly (*p* < 0.001) increased fruit juice pH while 2% PA showed no significant (*p* > 0.05) effect compared to no spray and water treatment (Fig. 5A). Moreover, fruit juice TDS and salinity were significantly (*p* < 0.001) higher in the fruits of 1% and 2% PA-treated plants under half NPK rate but comparable to no spray-treated plants (Fig. 5B,C). The 0.5% and 2% PA under full NPK rate significantly (*p* < 0.001) increased both fruit juice TDS and salinity compared to no spray treatment (Fig. 5B,C). Nevertheless, 0.25% PA with full NPK rate significantly (*p* < 0.001) decreased fruit juice TDS and salinity compared to water treatment although not significantly (*p* > 0.05) different from the no spray treatment (Fig. 5B,C). Likewise, 1% and 2% PA combined with half NPK rate considerably increased fruit juice EC by *ca*. 0.17 and 0.18 folds respectively compared to water treatment but was comparable to that of the no spray treatment (Fig. 5D). Furthermore, 0.5% and 2% PA with full NPK rate significantly (*p* < 0.001) improved fruit juice EC compared to no spray treatment but was not different from water treatment (Fig. 5D). However, 0.25% PA had no effect on fruit juice EC, irrespective of NPK rate (Fig. 5D).

### Leaf and fruit tissue phytochemical analysis

Leaf carotenoid content was significantly (*p* < 0.001) increased in 0.5% PA-treated plants under full NPK rate by *ca*. 0.33 and 0.19 folds compared to no spray and water treatment, respectively (Table 2). Similarly, 0.5% PA in addition to half NPK rate significantly (*p* < 0.001) enhanced fruit carotenoid content by *ca.* 0.30 and 0.13 folds compared to no spray and water treatment, respectively, but was not significantly (*p* > 0.05) different from that of the 2% PA (Table 3). On the contrary, 0.5% PA combined with full NPK rate significantly (*p* < 0.001) reduced fruit carotenoid content by *ca.* 0.17 and 0.15 folds compared to no spray and water treatment respectively (Table 3). Overall, leaf carotenoid content was *ca.* 26.31 folds higher than fruit carotenoid content (Table S3). Both leaf and fruit protein contents were significantly (*p* < 0.001) affected by PA and NPK interaction (Tables 2, 3). In general, total leaf protein content was significantly (*p* < 0.001) higher (*ca.* 1.19 folds) than fruit total protein (Table S3). Among the applied treatments, 2% PA with half NPK rate enhanced leaf protein content by *ca.* 0.13 and 0.12 folds compared to no spray and water treatment respectively (Table 2). However, 0.25% PA combined with full NPK rate markedly reduced leaf protein content. Besides, 2% PA with full NPK rate recorded the highest fruit protein content (*ca*. 0.40 and 0.67 folds) followed by 1% PA with full NPK rate (*ca.* 0.33 and 0.59 folds) compared to no spray and water treatment respectively (Table 3). Leaf and fruit sugar contents were significantly (*p* < 0.001) affected by PA and NPK interaction (Tables 2, 3). Relative tissue analysis revealed that fruit sugar content was significantly (*p* < 0.001) increased by *ca.* 1.48 folds compared to leaf sugar content (Table S3). All the PA treatments with full NPK rate considerably reduced leaf sugar contents which 0.5% PA recorded the lowest. Contrarily, 0.5% PA with half NPK rate increased leaf sugar content by *ca.* 0.26 folds compared to no spray treatment but not different from water treatment (Table 2). Besides, 2% PA with full NPK rate recorded the highest (*ca.* 0.32 and 0.15 folds) fruit sugar content compared to no spray and water treatment (Table 3).

**Table 2:** Phytochemical composition and ROS scavenging activities of tomato ‘Scotia’ leaves sprayed with pyroligneous acid (PA) under varying NPK rates.

PA	NPK rate	Carotenoid (mg g^−1^ FW)	Protein (mg g^−1^ FW)	Sugar (mg glucose g^−1^ FW)	Phenolics (mg GAE g^−1^ FW)	Flavonoid (µg quercetin g^−1^ FW)	DPPH (%)
0.25%	Full	64.21 g	2.00 f	6.86 ef	604.69d e	820.55 g	55.57 f
	Half	86.40 b	2.41 bcd	7.69 d	606.55 d	1030.91 bc	48.35 g
0.5%	Full	89.52 a	2.31 cde	6.74 f	529.74 g	899.81 e	42.56 h
	Half	79.26 cd	2.18 ef	9.01 b	580.66 f	1085.57 a	71.18 b
1%	Full	83.93 b	2.52 b	7.11 e	602.49 de	755.51 h	64.11 cd
	Half	71.05 fg	2.43 bcd	7.16 e	691.98 a	944.17 d	82.76 a
2%	Full	86.18 b	2.29 de	8.53 bc	460.21 h	1079.25 a	66.03 c
	Half	74.37 ef	2.64 a	7.96 cd	685.04 ab	931.02 d	72.49 b
No spray	Full	67.23 g	2.32 cde	8.82 b	657.47 c	1059.24 ab	58.62 ef
	Half	76.78 cde	2.33 cde	7.17 e	593.52 def	858.10 f	55.51 f
Water	Full	75.14 def	2.44 bc	9.95 a	592.51 ef	870.92 f	61.42 de
	Half	79.85 c	2.37 cd	8.37b c	674.05 b	1028.33 c	61.10 de

Sources of Variation		*p*-value					
PA		< 0.001	< 0.001	< 0.001	< 0.001	< 0.001	< 0.001
NPK		0.043	0.008	0.560	< 0.001	< 0.001	< 0.001
PA x NPK		< 0.001	< 0.001	< 0.001	< 0.001	< 0.001	< 0.001

The different letters indicate significant (*p* < 0.05) difference according to Fisher’s least significant difference (LSD) post hoc test.

**Table 3:** Phytochemical composition and ROS scavenging activities of tomato ‘Scotia’ fruit from plants sprayed with pyroligneous acid (PA) under varying NPK rates.

PA	NPK rate	Carotenoid (mg g^−1^ FW)	Protein (mg g^−1^ FW)	Sugar (mg glucose g^−1^ FW)	Phenolic (mg GAE g^−1^ FW)	Flavonoid (µg quercetin g^−1^ FW)	DPPH (%)
0.25%	Full	2.88 cde	1.16 c	20.29 d	134.91 d	57.92 e	24.05 i
	Half	2.51 fg	1.23 bc	13.43 f	106.64 g	59.28 e	25.50 h
0.5%	Full	2.38 g	1.14 c	21.21 c	259.46 a	58.58 e	29.46 f
	Half	3.39 a	1.13 cd	13.47 f.	152.43 c	66.39 d	30.68 e
1%	Full	2.70 defg	1.30 ab	12.28 g	67.80 h	26.87 f	32.95 b
	Half	2.78 def	1.04 de	21.77 c	181.77 b	94.37 b	33.14 b
2%	Full	3.08 bc	1.37 a	26.46 a	129.82 de	81.86 c	34.17 a
	Half	3.29 ab	1.13 cd	22.93 b	164.59 bc	108.95 a	31.82 c
No spray	Full	2.85 cde	0.98 e	20.14 d	158.03 c	70.17 d	31.01 de
	Half	2.61 efg	0.77 f	19.04 e	112.24 f	66.13 d	30.91 de
Water	Full	2.79 def	0.82 f	22.98 b	167.67 bc	104.84 a	28.57 g
	Half	3.00 bcd	0.78 f	19.44 e	122.96 e	21.41 g	31.30 d

Sources of Variation		*p*-value					
PA		< 0.001	< 0.001	< 0.001	< 0.001	< 0.001	< 0.001
NPK		0.016	< 0.001	< 0.001	< 0.001	0.663	0.001
PA x NPK		< 0.001	< 0.001	< 0.001	< 0.001	< 0.001	< 0.001

The different letters indicate significant (*p* < 0.05) difference according to Fisher’s least significant difference (LSD) post hoc test.

PA with NPK fertilizer interaction significantly (*p* < 0.001) affected total phenolic and flavonoid contents (Tables 2, 3). Leaf total phenolic and flavonoid contents were significantly (*p* < 0.001) increased by *ca.* 3.31 and 12.97 folds respectively compared to the tomato fruits (Table S3). The 1% PA combined with half NPK rate recorded a significantly (*p* < 0.001) higher (i.e., *ca.* 0.17 and 0.3 folds) leaf phenolic content while the 2% PA with full NPK rate recorded the least compared to no spray and water treatment (Table 2). Conversely, the 0.5% PA with full NPK rate significantly (*p* < 0.001) increased fruit phenolic content by *ca*. 0.65 and 0.55 folds while the 1% PA with full NPK rate recorded the least compared to no spray and water treatment respectively (Table 3). Furthermore, leaf flavonoid content was significantly (*p* < 0.001) increased with 0.5% PA under half NPK rate compared to no spray and water alone treatments (Table 2). The flavonoid content of the 0.5% PA-treated leaves was comparable to that of 2% PA and no spray treatments with half NPK rate. Nevertheless, the flavonoid content of 2% PA-treated leaves was significantly (*p* < 0.001) higher (*ca*. 0.05 folds) than that of water treatment (Table 2). Tomato fruit flavonoid content was significantly (*p* < 0.001) increased by *ca*. 0.65 and 4.09 folds following the application of 2% PA and half NPK rate compared to no spray and water treatment respectively (Table 3). Nevertheless, PA application with full NPK rate reduced fruit flavonoid content compared to water treatment but not the no spray treatment (Table 3). The ROS scavenging activity of leaf and fruit tissue extracts using DPPH scavenging activity showed a significant effect (*p* < 0.001) of PA and NPK fertilizer interaction (Tables 2, 3). The total DPPH scavenging activity of leaf tissues was significantly (*p* < 0.001) higher (*ca*. 1.03 folds) than that of the fruit (Table S3). All PA treatments with half NPK rate except for 0.25% PA significantly (*p* < 0.001) increased leaf DPPH scavenging activity by up to 0.49 and 0.36 folds compared to no spray and water treatment, respectively (Table 2). Likewise, a significantly (*p* < 0.001) high fruit DPPH scavenging activity (*ca*. 0.10 and 0.20 folds) was noticed in 2% PA-treated plants in combination with full NPK rate, whereas 0.25% PA recorded the least fruit DPPH scavenging activity compared to no spray and water treatments (Table 3).

### Fruit mineral element composition

Fruit elemental composition was highly influenced by PA and NPK fertilizer interaction (Table 4). Fruit N content was increased by up to *ca*. 0.10 folds in 0.25%, 0.5% and 1% PA-treated plants in combination with full NPK rate compared to water-treated plants (Table 4).

**Table 4:** Tomato ‘Scotia’ fruit elemental composition in response to pyroligneous acid (PA) under varying NPK rates.

PA	NPK rate	Fruit mineral element										
		N (%)	Ca (%)	K (%)	Mg (%)	P (%)	Na (%)	B (mg L^−1^)	Cu (mg L^−1^)	Fe (mg L^−1^)	Mn (mg L^−1^)	Zn (mg L^−1^)
0.25%	Full	1.62	0.17	2.68	0.14	0.41	0.03	11.39	5.47	43.35	26.44	22.91
	Half	1.33	0.15	2.72	0.13	0.40	0.04	11.87	5.18	40.06	21.82	17.63
0.5%	Full	1.60	0.14	2.94	0.14	0.44	0.03	11.91	5.56	40.68	24.47	21.31
	Half	1.38	0.18	2.82	0.14	0.42	0.03	11.62	ND	100.07	20.54	17.6
1%	Full	1.52	0.19	2.64	0.14	0.39	0.03	10.65	5.38	57.36	25.62	22.17
	Half	1.27	0.20	3.09	0.14	0.42	0.03	12.33	5.01	107.47	24.22	24.35
2%	Full	1.46	0.16	2.98	0.13	0.43	0.03	12.36	5.69	309.67	25.64	18.72
	Half	1.42	0.20	2.99	0.15	0.42	0.03	11.99	ND	267.86	24.9	20.28
No spray	Full	1.72	0.17	2.61	0.15	0.44	0.03	11.58	7.07	42.68	27.62	18.86
	Half	1.24	0.22	2.96	0.14	0.41	0.03	12.41	ND	43.69	25.42	18.53
Water	Full	1.47	0.18	2.83	0.13	0.41	0.02	11.43	5.47	59.99	18.04	18.46
	Half	1.12	0.22	2.61	0.13	0.36	0.05	11.58	ND	128.67	23.56	15.36
Mean		1.43	0.18	2.82	0.14	0.41	0.03	11.76	5.60	103.46	24.02	19.68
CV (%)		12.12	13.29	5.93	4.30	5.21	19.99	4.26	11.24	88.90	11.22	13.09

*ND* Not determined.

Fruit Ca content was slightly increased in 1% PA-treated plants with full NPK while 0.5% recorded the least Ca content (Table 4). Also, high fruit K content was noticed in fruits of the 1% PA-treated plants followed by 2% PA with half NPK rate compared to no spray and water treatment (Table 4). Besides, PA and NPK application had no obvious effect on fruit Mg, P and Na content. Fruit B content was increased by *ca.* 0.08 folds in fruits of 2% PA with full NPK rate compared to no spray and water treatment. Fruit Cu content was reduced in fruits of PA with full NPK rate treated plants compared to no spray treatment but was slightly increased with 1% and 2% PA when compared to water treatment. Moreover, 2% PA markedly increased fruit Fe content by *ca*. 6.26 and 4.16 folds under full NPK rate and by *ca*. 5.13 and 1.08 folds under half NPK rate compared to no spray and water treatment respectively. However, 0.25% PA reduced fruit Fe content irrespective of NPK rate. Fruit Mn content was marginally reduced with PA application under full NPK rate compared to no spray treatment but increased considerably with all PA treatments compared to water treatment. Under half NPK rate, fruit Mn content was not altered with PA application compared with no spray and water treatments. Furthermore, 1% PA with half NPK rate enhanced fruit Zn content by *ca.* 0.32 and 0.59 folds followed by 2% PA compared to no spray and water treatments, respectively.

### Association between morpho-physiological properties, productivity and phytochemicals

**Figure 6:**
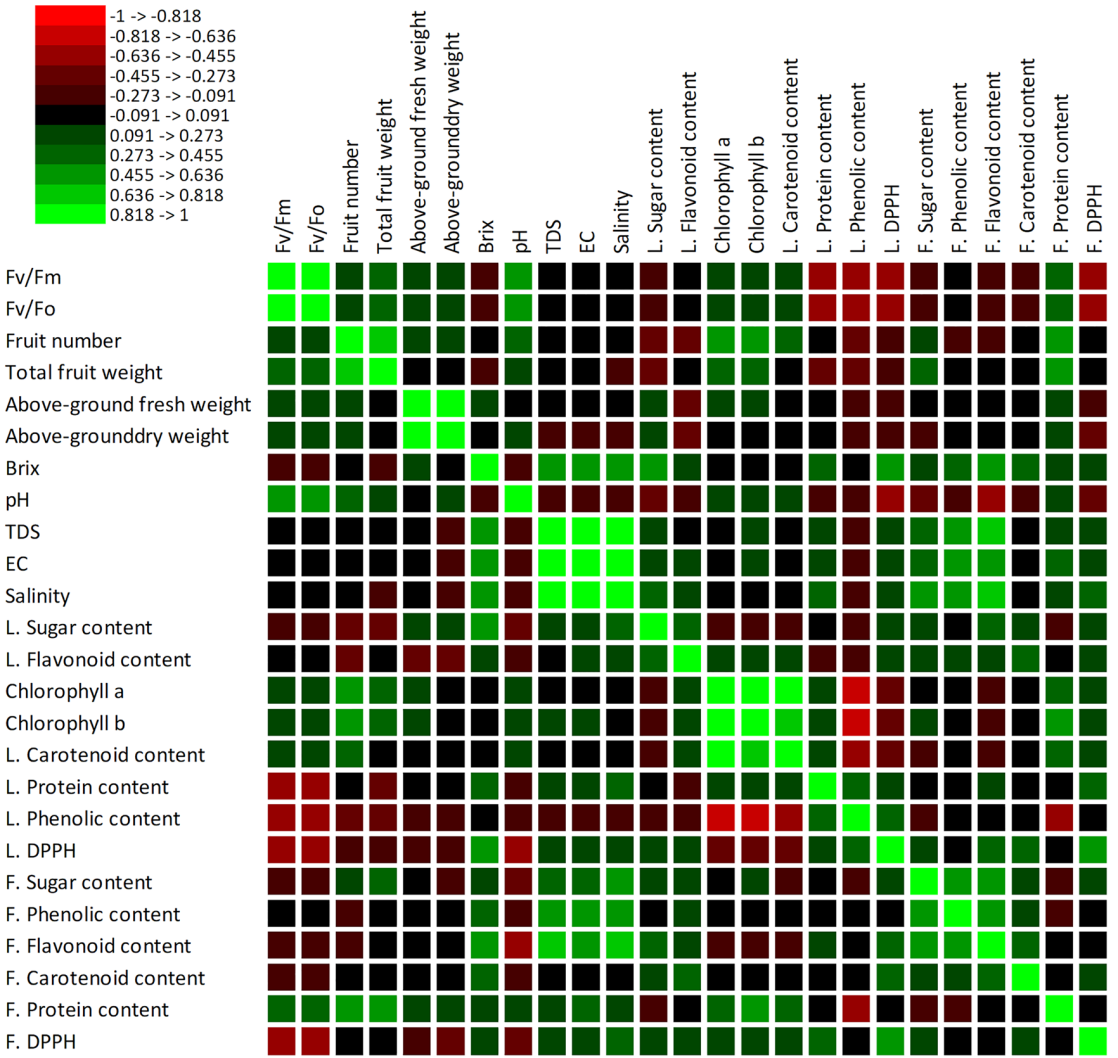
Pearson correlation matrix among the morpho-physiological, yield, quality, and phytochemical properties of tomato plants in response to PA and NPK combination. The red colour represents a strong negative association, and the green colour represents a strong positive association.

**Figure 7:**
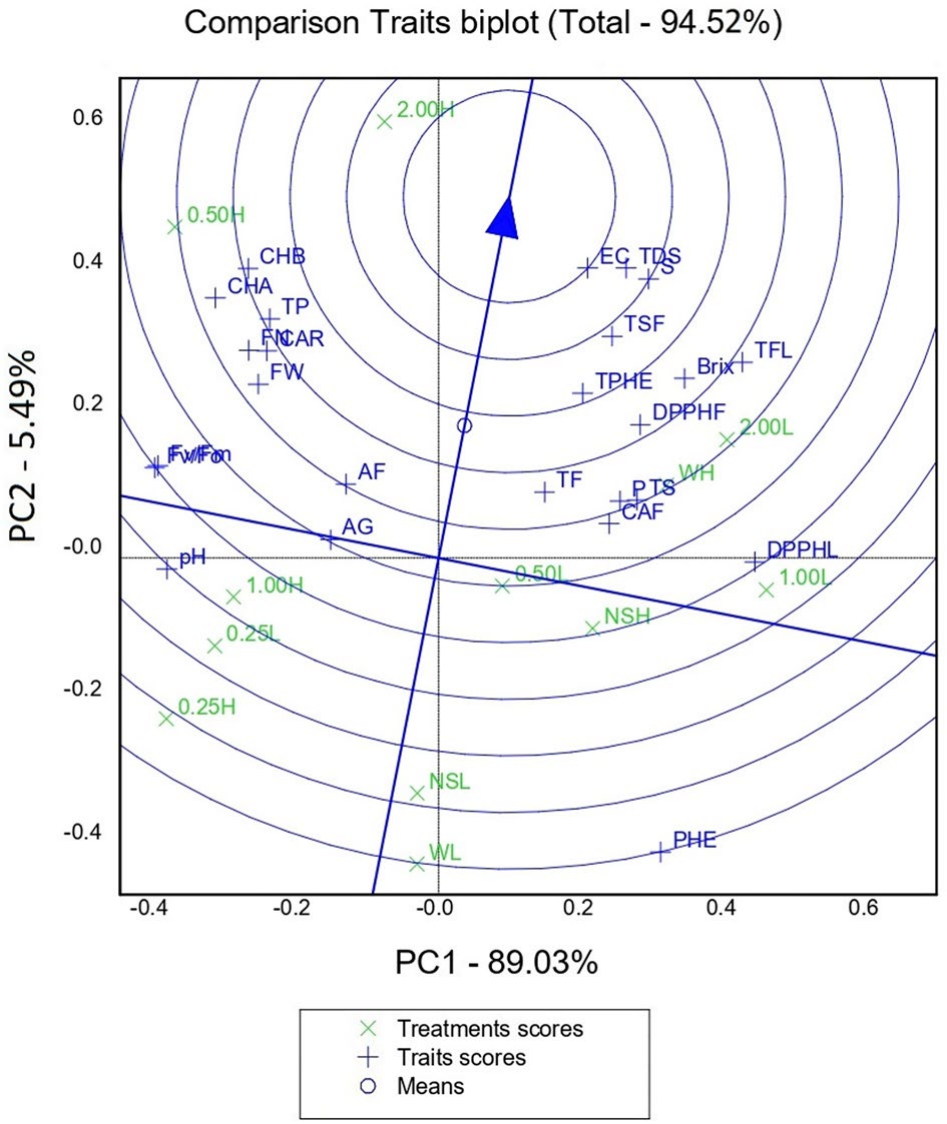
A two-dimensional principal component (2-D PCA) analysis biplot shows relationships amongst tomato plants' morpho-physiological, yield, quality, and phytochemical properties in response to PA and NPK combination. Projection of the variables in the principal component (PC1 and PC2) explained a total of 95.52% of the variations in the dataset. Variables that are closely located are not different compared to variables located at a distance within a quadrant or between quadrants. Total fruit number, FN; Total fruit weight, FW; Above-ground fresh weight, AF; Above-ground dry weight, AG; Fruit juice Brix, Brix; fruit juice pH, pH; fruit juice total dissolved solids, TDS; fruit juice electrical conductivity, EC; fruit juice salinity, S; Leaf total sugar, TS; Leaf total flavonoid content, TF; Chlorophyll a, CHA; Chlorophyll b, CHB; Leaf carotenoid, CAR; Leaf total protein, P; Leaf total phenolics, PHE; Leaf DPPH, DPPHL; Fruit total sugar, TSF; Fruit total phenolic content, TPHE; Fruit total flavonoid content, TFL; Fruit carotenoid, CAF; Fruit total protein, TP; Fruit DPPH, DPPHF. No spray + Full NPK rate, NSH; No spray + Half NPK rate, NSL; Water + Full NPK rate, WH; Water + Half NPK rate, WL; 0.25% PA + Full NPK rate, 0.25H; 0.25% PA + Half NPK rate, 0.25L; 0.5% PA + Full NPK rate, 0.5H; 0.5% PA + Half NPK rate, 0.5L; 1% PA + Full NPK rate, 1H; 1% PA + Half NPK rate, 1L; 2% PA + Full NPK rate, 2H; 2% PA + Half NPK rate, 2L.

A two-dimension (2-D) principal component analysis and Pearson correlation coefficients (r) were used to further asses the association amongst the morpho-physiological, yield and phytochemical properties of tomato plants in response to PA and NPK fertilizer interaction (Figs. 6, 7, Table S4). The correlation analysis showed that *Fv*/*Fm* had a significant (*p* < 0.001) positive association with *Fv*/*Fo*, fruit weight, and fruit juice pH, but exhibited a significant (*p* < 0.01) negative correlation with leaf protein content, phenolic content and leaf and fruit DPPH. Similarly, total number of fruits had a significant (*p* < 0.01) positive correlation with total fruit weight, chlorophyll a and b and fruit protein content and a negative association with leaf sugar content. Total fruit weight had a significant (*p* < 0.01) negative association with leaf phenolic content but a positive association with fruit protein content. Above-ground fresh weight had a significant (*p* < 0.01) positive correlation with aboveground dry weight and a negative association with leaf flavonoid content (Fig. 6, Table S4). Likewise, fruit juice Brix content exhibited a significant (*p* < 0.01) positive correlation with fruit juice TDS, EC, salinity, leaf sugar, leaf DPPH and fruit flavonoid contents (Fig. 5, Table S4). However, fruit juice pH showed a significant (*p* < 0.01) negative association with leaf DPPH, fruit flavonoid and fruit DPPH (Fig. 6). Fruit juice TDS had a significantly (*p* < 0.01) positive association with fruit juice EC, salinity, fruit total sugar, phenolics and flavonoid contents. Similar to TDS, EC had a significantly (*p* < 0.01) strong positive association with fruit juice salinity while both fruit juice EC and salinity had a significantly (*p* < 0.01) positive association with fruit total sugar, phenolics and flavonoid contents (Fig. 6, Table S4). Moreover, chlorophyll a exhibited a significant (*p* < 0.01) association with chlorophyll b and total leaf carotenoid. Both chlorophyll a and b and leaf carotenoid contents had a significant (*p* < 0.01) negative and positive correlation with leaf total phenolic and DPPH content, and total fruit protein respectively (Fig. 6, Table S4). Also, both leaf total protein and leaf DPPH had a significant (*p* < 0.01) positive association with fruit DPPH while leaf total phenolic content had a negative correlation with fruit protein. Additionally, fruit total sugar content showed a significant (*p* < 0.01) positive correlation with fruit total phenolic and flavonoid content while fruit total phenolic content exhibited a significant (*p* < 0.01) positive association with fruit flavonoid (Fig. 6, Table S4).

The 2D PCA biplot explained *ca*. 95% of the total variations in the dataset (Fig. 7). Response variables and treatments that are closer to the arrow had the maximum effect on the overall plant productivity while those that are distant from the arrow exhibit a minimum effect. The results revealed that fruit juice EC, TDS, salinity, Brix, fruit total phenolic content, leaf and fruit total sugar, fruit total flavonoid content, fruit total protein, leaf and fruit carotenoid content, fruit DPPH, chlorophyll a and b, fruit number and total fruit weight were strongly influenced by PA and NPK fertilizer treatments (Fig. 7). However, fruit juice pH and leaf total phenolics were weakly affected by the treatments. Additionally, chlorophyll fluorometric indices, aboveground fresh and dry weights, leaf total protein, leaf total sugar, leaf total flavonoid and leaf DPPH were moderately affected by the treatment application (Fig. 7). Moreover, 0.5% PA and 2% PA combined with full NPK rate had the maximum positive effect on the overall performance of tomato plants while no spray and water treatments with half NPK rate had the least effect on the growth and productivity of the greenhouse-grown tomato plants (Fig. 7).

## Discussion

The use of PA as a biostimulant to boost plant productivity and phytochemical properties in edible plant parts has spiked the interest of farmers and researchers. This is motivated by the quest for environmentally friendly strategies for sustainable crop production. The results of this study clearly revealed that foliar application of 0.25% and 0.5% PA increased *Fv*/*Fo* and *Fv/Fm* but were higher when combined with the full NPK rate which is recommended for tomato production. Chlorophyll fluorescence has been widely used to determine the photosynthesis performance of plants^41^. Previous studies revealed that PA application increased *Fv*/*Fm* values in rice (*Oryza sativa*)^16^, tobacco (*Nicotiana tabacum*)^19^, and lettuce (*Lactuca sativa*)^18^.

Chlorophyll plays a crucial role in photosynthesis and the overall productivity of crops^42^ and its level in plant leaves reflects the strength of the photosynthetic process^42,43^. In this study, 2% PA with full NPK rate increased chlorophylls a and b, suggesting that PA and NPK applications exhibited a synergistic effect on plant photosynthetic capacity. This result is consistent with previous studies where a similar increase in chlorophyll content with different PA concentrations have been reported^13,16,18,19,39^. Besides, the PA used in this study was previously revealed to contain high levels of N, K and Ca as well as other bioactive compounds^11^. In plants, N is a key modulator of nitrogenous compounds biosynthesis including chlorophyll and proteins, K is critical in enzyme activation, stomatal regulation and photosynthesis while Ca facilitate nutrient uptake and cell elongation^44,45^. Moreover, high polyphenol has been correlated with high chlorophyll content in leaf tissues due to the former’s significant contribution in reducing oxidative stress-induced photorespiration^46^. PA is known to contain high levels of polyphenols, which have been suggested to mediate the increased chlorophyll content in lettuce^18^. Hence, the increase in chlorophyll parameters with foliar PA application can be attributed to its rich N, K, Ca and bioactive compounds that can stimulate root nutrient uptake, reduce oxidative stress-induced photorespiration, and enhance the biosynthesis of chlorophyll.

The impact of PA on crop yield has been reported extensively by several authors^17,20–23,31^. Similarly, our results showed that 2% PA irrespective of NPK fertilizer rate increased the total number of fruits and fruit weight followed by 0.5% PA combined with full NPK rate. Although the positive effect of NPK cannot be neglected, a similar increase in fruit number and weight have been reported in tomato^20,21,24^ and sweet pepper (*Capsicum annuum*)^47^. Additionally, the high total fruit weight of 2% PA with full NPK rate compared to the half NPK rate can be attributed to the differences in the sizes of marketable fruit. Although the exact mechanism of the PA-mediated increase in fruit yield is still under investigation, it can be suggested that the increase in fruit yield could be due to increased chlorophyll content which indicates high photosynthetic translocation of assimilates into the increased number of fruits^42,48^. This was revealed in the correlation analysis that total fruit weight and number of fruits had a significant association with chlorophyll a and b content. Furthermore, PA is a complex mixture of numerous bioactive compounds including alkanes, phenolics, esters, organic acids and alcohol^10^. Previous study revealed that karrikins present in PA behave similarly to other phytohormones and interact with gibberellins, ethylene and cytokinin to regulate plant growth and productivity^32^. This indicates that PA might have induced and cooperated with the functions of enzymes and hormones to increase plant yield. Additionally, the reduction of fruit number and total fruit weight of both no spray and water spray treatments in the half NPK rate could be due to reduced fruit sets and/ or plant nutrient assimilation.

Chemical properties and phytochemical composition of edible plant portions are important parameters considered in the marketability of crops. This study showed that 2% PA with half NPK increased fruit Brix content while both fruit TDS and salinity were higher in 1% PA-treated plants under half NPK rate, and 0.5% and 2% PA-treated plants irrespective of NPK rate. Brix content is commonly used to determine the quality of tomato concentrate and correlates with the fruit TDS^26^. Consistently, PA and other biostimulants were reported to increase the Brix of tomato fruits^21,26^. This indicates that PA can be used to enhance tomato fruit flavour and taste^26^. Moreover, our correlation analysis confirmed that fruit salinity content exhibits a significantly positive association with its Brix, TDS and EC. Moreover, the high fruit TDS, EC and salinity in no spray treatments can be attributed to low dilution effect due to reduced fruit sizes as reported by Marles^49^. Intriguingly, tissue sugar analysis revealed that the total fruit sugar content was higher than that of the leaf tissue. While PA application alone reduced leaf sugar content, 2% PA combined with full NPK rate recorded the highest fruit sugar content. It is well known that sugar production begins with leaf photosynthesis and the assimilate is translocated to the developing fruit in the case of tomato. Sugar distribution to fruits depends on several bioprocesses including photosynthesis rate, carbohydrate absorption and metabolism in sink organ, and phloem loading and unloading^50,51^. The overall reduction in leaf tissue sugar content suggests that PA application increased photo-assimilation and translocation of sugar into the fruits. Fedeli et al.^39^ reported a similar reduction of sugar content in lettuce leaves following PA treatment. Also, some studies have shown an increase in fruit sugar content in tomato^21^ and rock melon^23^. It is therefore plausible to indicate that the increased fruit sugar content can be linked to the high photosynthetic performance of PA-treated plants.

Moreover, leaves of some crops have been reported to contain a high content of phytochemicals (e.g., flavonoids and phenolics) than fruit tissues^52,53^. An increase in phytochemicals in leaf tissues following PA application has been reported in lettuce^39^ and strawberry (*Fragaria* × *ananassa*)^25^. The increase in tomato leaves phytochemicals in the present study can be attributed to the high phenolic and organic acid content in PA as previously reported by Loo, et al.^33^. This finding indicates that PA can be adopted as a strategy to increase the nutritional and health value of leafy vegetables. Generally, tomato fruits are deemed as a good reservoir of phytochemicals including carotenoids, phenolics and flavonoids^2^. In this study, 0.5% PA with half and full NPK rates enhanced fruit carotenoid and phenolics contents respectively, while 2% PA increased fruit flavonoid content. Accordingly, several studies have reported that PA application increased phenolics and flavonoid contents in tomato fruits^21,24^.

Carotenoids are lipophilic antenna pigments in photosynthesis and are crucial for maintaining good human health^54^. The increased carotenoid content with PA application can be attributed to the stimulation of physiological and molecular pathways involved in nitrogen metabolism^54,55^. Besides, these compounds are known to exhibit high antioxidant properties by scavenging reactive oxygen species (ROS) radicals and thereby, protecting cells against oxidative stress^2,3^. High ROS scavenging activity was recorded following 2% PA treatment. The increase in DPPH activity was highly expected and may be correlated with enhanced phytochemical content as previously reported^17,24^. Additionally, it was reported that the high phenolic content of PA exhibited high anti-lipid peroxidation and antioxidant capacity^11,12,33^. These findings suggest that foliar PA application with NPK fertilizer combination may be considered as an environmentally friendly plant growth promotion technique to increase fruit nutritional quality and promote health benefits.

Fruit mineral elements are crucial for numerous plant physiological and biochemical processes, and constitute a relatively small portion of dry fruit tissues^56,57^. This study showed that both 0.25% and 0.5% PA combined with full NPK rate enhanced fruit N content while 0.5% PA increased fruit P. The 1% PA with half NPK increased fruit K and Zn content whereas 2% PA increased fruit K and Fe irrespective of NPK rate. Additionally, 2% PA with half NPK rate increased fruit Mg content. According to Bhat, et al.^57^, the uptake of these nutrients by plants is dependent on the soil constituents and/or supplied when plants are foliar sprayed with fertilizers or biostimulants. Besides, the increase in N, P and K content can be attributed to increased soil supply of NPK fertilizer rate. Also, increased nutrient uptake is promoted by increased primary or secondary metabolism and enhanced enzyme activities for physiological growth^57^. These results agree with previous studies where PA and other biostimulants were reported to increase fruit mineral elements in tomato^21,56^. Although the mechanism by which PA increase these elements remains unknown, it can be speculated that the bioactive composition of PA could: (1) promote sink strength for continuous mineral element flow and accumulation^58^; (2) stimulate genes encoding nutrient transporters in cell membranes; and (3) stimulate root system to facilitate intense nutrient uptake and translocation^14^.

## Materials and methods

### Plant material and growing condition

This study was carried out from August to December 2021 and repeated from February to June 2022 in the Department of Plant, Food, and Environmental Sciences, Faculty of Agriculture greenhouse. Tomato (*Solanum lycopersicum* “Scotia”) seeds were purchased from Halifax seeds (Halifax, Canada). Seeds were sterilized with 10% NaClO for 10 min, thoroughly washed three times with sterile distilled water (ddH_2_O) followed by 70% ethanol sterilization for 5 min, and subsequently washed 5 times with sterile distilled water. The PA was derived from white pine biomass and prepared by Proton Power Inc (Lenoir City, USA). The seeds were sown in 32-cell packs containing Pro-Mix® BX (Premier Tech Horticulture, Québec, Canada). The germinated seedlings were grown for 4 weeks in a growth chamber with a day/night temperature regime of 24/22 °C, 16/8 h d^−1^ illumination at a light intensity of 300 μmol m^−2^·s^−1^ and relative humidity of 70%. The seedlings were transplanted into 11.35 L-plastic pots containing Pro-Mix® BX medium and acclimatized for a week under greenhouse conditions before treatment application. The greenhouse condition was day/night temperature of 28 °C/20 °C, 16/8 h d^−1^ photoperiod, relative humidity of 70% and supplemental lighting provided by a 600 W HS2000 high-pressure sodium lamp with NAH600.579 ballast (P.L. Light Systems, Beamsville, Canada).

### Experimental treatment and design

The study was arranged in a 4 × 2 factorial completely randomized design with four replications. The treatment factors were varying PA concentrations (0%, 0.25%, 0.5%, 1% and 2% PA: ddH_2_O) and water-soluble fertilizer (NPK (15:15:30)) (Botaflora, Groupe Bmr, Boucherville, Québec, Canada) applied at a full rate (2.5 g/L) and half rate (1.25 g/L). The full rate of 2.5 g/L was based on manufacturers’ recommendations for tomato production. The full rate consisted of 0.63, 0.28, 1.03 g/L of N, P and K respectively, and half of the recommended rate contained 0.32, 0.14, 0.52 g/L of N, P and K respectively. PA was applied bi-weekly as foliar sprayed using an 8-L capacity sprayer until the leaves started dripping. The liquid NPK fertilizer was applied as a soil drench every 21 days. The potted plants were rearranged weekly on the bench to offset unpredictable occurrences due to variations in microclimate.

### Plant physiology and yield measurements

Chlorophyll fluorescence indices were determined from five leaves per plant at 50 days after transplanting (DAT) using a chlorophyll fluorometer (Optical Science, Hudson, NH, USA). Briefly, leaves were first dark adapted for 25 min before the initial fluorescence yield (F_o_) was measured, followed by the maximum chlorophyll fluorescence (F_m_) emitted during a saturating light pulse. Variable chlorophyll fluorescence (F_v_) was calculated as F_v_ = F_m_ − F_o_. Fluorometric parameters including maximum quantum efficiency (F_v_/F_m_) and potential photosynthetic capacity (F_v_/F_o_) were determined at each saturating pulse^41^. At 80 DAT, ripe fruits were harvested and the total fresh weight per plant was recorded using an XT portable balance (Ohaus navigator®, ITM Instruments Inc., Sainte-Anne-de-Bellevue, QC, Canada). Fruit equatorial and polar diameters were measured with a digital Vernier caliper (Mastercraft®, Ontario, Canada). The harvested fruits were graded according to the Canadian Food Inspection Agency (CFIA) grade compendium for fresh vegetables^40^. After harvest, the total above-ground of the whole plant (i.e., leaves and shoot) was weighed for above-ground fresh weight and followed by oven-drying at 65 °C for 72 h for above-ground dry weight determination.

### Fruit quality and leaves tissue biochemical analysis

During the first fruit harvest, leaf samples (15 leaves/treatment) were collected, flash-frozen in liquid nitrogen and stored in a − 80 °C freezer. Ten ripe fruits of average size and colour were randomly selected, and surface sterilized with 70% ethanol. The fruit pericarp (consisting of the epidermis) was excised from the longitudinal section using a sterilized scalpel blade, flash-frozen in liquid nitrogen and stored in a − 80 °C freezer for biochemical analysis. The remaining portion of the fruit was kept at − 20 °C until further quality analysis. The fruit quality analyses were performed as described by Ofoe et al.^21^. The frozen fruits were thawed at room temperature and hand-squashed in a clear Ziplock bag. Total soluble solids (TSS) of the fruit juice was determined using a handheld refractometer (Atago, Tokyo, Japan) and expressed as degree Brix (^o^Brix). Fruit juice pH, salinity, total dissolved solids (TDS), and electric conductivity (EC) were measured with a 3-in-1 pH meter (Extech Instrument, Taiwan). The complete macro- and micro-nutrient contents of the fruits were determined using inductively coupled plasma mass spectrometry (PerkinElmer Perkin Elmer 2100DV, USA) at the Nova Scotia Department of Agriculture Laboratory, Truro, NS^59^.

#### Chlorophyll a and b, and carotenoid content

Chlorophyll a and b of the leaves, and carotenoid content of leaves and fruits were determined as described by Lichtenthaler^60^ with little modification. A 0.2 g of both ground leaf tissues and fruit pericarp were separately homogenized in 2 mL of 80% acetone. The mixture was centrifuged at 15,000×*g* for 15 min and the absorbance of the supernatant was measured at 646.8, 663.2, and 470 nm using a UV–Vis spectrophotometer (Jenway, Staffordshire, UK) against 80% acetone as blank. Leaf chlorophyll a and b, and the total carotenoid content of both leaves and fruits were expressed as a µg/g FW of the sample.

#### Leaf and fruit total soluble sugar contents

Total soluble sugar content was estimated according to the phenol–sulphuric acid method described by Dubois et al.^61^. 0.2 g of ground leaf and fruit tissues were separately mixed in 10 mL of 90% ethanol. The mixture was vortexed for 3 min and incubated in a water bath at 60 °C for 60 min. The final volume of the mixture was adjusted to 5 mL with 90% ethanol and centrifuged at 12,000 × *g* for 3 min. A 1 mL aliquot of the supernatant was transferred into a thick-walled glass test tube containing 1 mL of 5% phenol and mixed thoroughly. A 5 mL of concentrated sulphuric acid was added to the reaction mixture, vortexed for 20 s, and incubated in the dark for 15 min. The mixture was cooled at room temperature and the absorbance was measured at 490 nm against a blank containing deionised water, phenol and sulphuric acid. Total sugar was calculated using a standard glucose curve and expressed as µg of glucose/g FW.

#### Leaf and fruit total phenolics

Total phenolics content (TPC) was determined by the Folin-Ciocalteu assay described by Ainsworth and Gillespie^62^ with little modification. 0.2 g of ground leaf and fruit tissues were separately homogenized in 2 mL of ice-cold 95% methanol and incubated in the dark at room temperature for 48 h. The mixture was centrifuged at 13,000×*g* for 5 min before adding 100 µL of the supernatant to 200 µL of 10% (v/v) Folin–Ciocalteau reagent. The mixture was vortexed for 5 min, mixed with 800 µL of 700 mM Na_2_CO_3,_ and incubated in the dark at 25 °C for 2 h. The absorbance of the supernatant was measured at 765 nm against a blank. TPC was calculated using a gallic acid standard curve and expressed as mg gallic acid equivalents per g fresh weight (i.e., mg GAE/g FW).

#### Leaf and fruit total flavonoid

Total flavonoid was estimated following the colorimetric method described by Chang et al.^63^. 0.2 g of ground leaf and fruit tissues were separately homogenized in 2 mL of ice-cold 95% methanol followed by centrifugation at 15,000×*g* for 10 min. 500 µL of the supernatant was added to a reaction mixture containing 1.5 mL of 95% methanol, 0.1 mL of 10% AlCl_3_, 0.1 mL of 1 M potassium acetate, and 2.8 mL of distilled water. The mixture was incubated at room temperature for 30 min and the absorbance was measured at 415 nm against a blank lacking AlCl_3_. Total flavonoid content was estimated using the quercetin standard curve and expressed as µg quercetin equivalents per g fresh weight (µg quercetin/g FW).

#### Leaf and fruit total protein content

For leaf and fruit total protein content, 0.2 g of ground leaf tissues and fruit pericarp were homogenized in 1.8 mL ice-cold extraction buffer (50 mM potassium phosphate buffer at a pH 7.0, 1% polyvinylpyrrolidone, and 0.1 mM EDTA). The homogenate was centrifuged at 15,000×*g* for 20 min at 4 °C. The supernatant (crude enzyme extract) was transferred to a new microfuge tube on ice and the protein content was measured at 595 nm after 5 min of mixing with Bradford’s reagent^64^. Protein content was estimated from a standard curve of bovine serum albumin (BSA) and expressed as mg BSA/g FW.

#### DPPH free radical scavenging capacity

The DPPH radical scavenging capacity was determined using the method described by Dudonne et al.^65^ with little modification. Briefly, 0.2 g of ground leaf tissue and fruit pericarp were homogenized in 1.5 mL pure methanol. The mixture was centrifuged at 12,000×*g* for 10 min and 100 µL of supernatant was added to 2.9 mL of 60 µM fresh DPPH methanolic solution. The mixture was vortexed and incubated in the dark at room temperature for 30 min. The absorbance of the reaction mixture was measured at 515 nm against methanol blank and the radical scavenging activity was calculated using the formula:

Inhibition (%) = [(A_B_ − A_S_)/A_B_] × 100%; where A_B_ is the absorbance of the blank sample and A_S_ is the absorbance of the sample.

### Statistical analysis

All data collected from the experiments were subjected to a two-way analysis of variance (ANOVA) using Minitab (Minitab 19 Statistical Software, USA). Means with significant differences were separated using the Fisher test at α = 0.05. A multivariate analysis including Pearson correlation and two-dimensional principal component analysis (PCA) biplot was performed using XLSTAT software (Version 2022.1, Lumivero, Colorado, USA).

## Conclusion

The present study clearly showed a synergistic effect of foliar PA application and soil-drenched NPK in enhancing tomato productivity and phytochemical composition. Foliar application of 0.25% and 0.5% PA increased chlorophyll fluorometric parameters, while 2% PA with full NPK rate increased leaf chlorophyll content. Also, 2% PA increased the total number of fruits irrespective of NPK rate but increased the overall fruit weight and marketable number of fruits with full NPK rate. The PA-treated plants accumulated higher phytochemical content in their leaves than in fruit tissues except for total sugar. The 2% and 0.5% PA with various NPK combinations enhanced fruit phytochemical contents. Besides, the synergistic effect of PA and fertilizer increased fruit mineral elemental composition. Taken together, this study demonstrated that 2% PA with full NPK rate is the best treatment combination that can be adopted to increase the productivity and nutritional benefits of greenhouse-grown tomato plants and can be extended through further studies to improve the nutritional and health value of leafy vegetables. Also, further study is required to investigate the molecular basis of PA biostimulatory effect on plants. Furthermore, PA application in mainstream agriculture represents a novel natural product that can contribute to the achievement of the United Nations' sustainable development goals by enhancing the production of nutrient-dense vegetables, promoting sustainable crop production, and ending world hunger.

### Supplementary Information

Supplementary Information.

## Data Availability

The data for the findings of this study are available from the corresponding author upon reasonable request.

## References

[1] Heuvelink E (2018). Tomatoes.

[2] Chaudhary P, Sharma A, Singh B, Nagpal AK (2018). Bioactivities of phytochemicals present in tomato. J. Food Sci. Technol..

[3] Nowak D, Gośliński M, Wojtowicz E, Przygoński K (2018). Antioxidant properties and phenolic compounds of vitamin C-rich juices. J. Food Sci..

[4] Ye L (2020). Bio-organic fertilizer with reduced rates of chemical fertilization improves soil fertility and enhances tomato yield and quality. Sci. Rep..

[5] Pretty J, Bharucha ZP (2014). Sustainable intensification in agricultural systems. Ann. Bot..

[6] Fan Z (2014). Conventional flooding irrigation causes an overuse of nitrogen fertilizer and low nitrogen use efficiency in intensively used solar greenhouse vegetable production. Agric. Water Manag..

[7] Dudaš S (2016). The effect of biostimulant and fertilizer on “low input” lettuce production. Acta Botanica Croatica.

[8] Carvalho FP (2006). Agriculture, pesticides, food security and food safety. Environ. Sci. Policy.

[9] Tripathi AD, Mishra R, Maurya KK, Singh RB, Wilson DW (2019). The Role of Functional Food Security in Global Health.

[10] Grewal A, Abbey L, Gunupuru LR (2018). Production, prospects and potential application of pyroligneous acid in agriculture. J. Anal. Appl. Pyrol..

[11] Ofoe R, Gunupuru LR, Abbey L (2022). Metabolites, elemental profile and chemical activities of Pinus strobus high temperature-derived pyroligneous acid. Chem. Biol. Technol. Agric..

[12] Wei Q, Ma X, Zhao Z, Zhang S, Liu S (2010). Antioxidant activities and chemical profiles of pyroligneous acids from walnut shell. J. Anal. Appl. Pyrol..

[13] Wang H (2019). Effects of wood vinegar on cold resistance of rice seedlings under low-temperature stress. J. Northeast Agric. Univ..

[14] Wang Y (2019). Root proteomics reveals the effects of wood vinegar on wheat growth and subsequent tolerance to drought stress. Int. J. Mol Sci..

[15] Ofoe R (2022). Seed priming with pyroligneous acid mitigates aluminum stress, and promotes tomato seed germination and seedling growth. Plant Stress.

[16] Berahim Z (2014). Rice yield improvement by foliar application of phytohormone. J. Food Agric. Environ..

[17] Fedeli R (2022). Foliar application of wood distillate boosts plant yield and nutritional parameters of chickpea. Ann. Appl. Biol..

[18] Vannini A, Moratelli F, Monaci F, Loppi S (2021). Effects of wood distillate and soy lecithin on the photosynthetic performance and growth of lettuce (*Lactuca sativa* L.). SN Appl. Sci..

[19] Ye Y (2022). Wood vinegar alleviates photosynthetic inhibition and oxidative damage caused by *Pseudomonas syringae* pv. tabaci (Pst) infection in tobacco leaves. J. Plant Interact..

[20] Mungkunkamchao T, Kesmala T, Pimratch S, Toomsan B, Jothityangkoon D (2013). Wood vinegar and fermented bioextracts: Natural products to enhance growth and yield of tomato (*Solanum lycopersicum* L.). Sci. Hortic..

[21] Ofoe R, Qin D, Gunupuru LR, Thomas RH, Abbey L (2022). Effect of pyroligneous acid on the productivity and nutritional quality of greenhouse tomato. Plants.

[22] Zhu K (2021). Wood vinegar as a complex growth regulator promotes the growth, yield, and quality of rapeseed. Agronomy.

[23] Zulkarami B, Ashrafuzzaman M, Husni MO, Ismail MR (2011). Effect of pyroligneous acid on growth, yield and quality improvement of rockmelon in soilless culture. Aust. J. Crop Sci..

[24] Benzon HRL, Lee SC (2016). Potential of wood vinegar in enhancing fruit yield and antioxidant capacity in tomato. Korean J. Plant Resour..

[25] Kårlund A (2014). Polyphenols in strawberry (Fragaria× ananassa) leaves induced by plant activators. J. Agric. Food Chem..

[26] Maach M (2021). Application of biostimulants improves yield and fruit quality in tomato. Int. J. Vegetable Sci..

[27] Jung K-H (2007). Growth inhibition effect of pyroligneous acid on pathogenic fungus, *Alternaria mali*, the agent of alternaria blotch of apple. J. Biotechnol. Bioprocess Eng..

[28] Mourant D, Yang D-Q, Lu X, Roy C (2007). Anti-fungal properties of the pyroligneous liquors from the pyrolysis of softwood bark. Wood Fiber Sci..

[29] Chiwocha SDS (2009). Karrikins: A new family of plant growth regulators in smoke. Plant Sci..

[30] Dixon KW, Merritt DJ, Flematti GR, Ghisalberti EL (2009). Karrikinolide–A phytoreactive compound derived from smoke with applications in horticulture, ecological restoration and agriculture. Acta Hortic..

[31] Kulkarni MG, Ascough GD, Van Staden J (2008). Smoke-water and a smoke-isolated butenolide improve growth and yield of tomatoes under greenhouse conditions. Horttechnology.

[32] Van Staden J, Jager AK, Light ME, Burger BV (2004). Isolation of the major germination cue from plant-derived smoke. South Afr. J. Botany.

[33] Loo AY, Jain K, Darah I (2008). Antioxidant activity of compounds isolated from the pyroligneous acid, Rhizophora apiculata. Food Chem..

[34] Drobek M, Frąc M, Cybulska J (2019). Plant biostimulants: Importance of the quality and yield of horticultural crops and the improvement of plant tolerance to abiotic stress—A review. Agronomy.

[35] Lashari MS (2013). Effects of amendment of biochar-manure compost in conjunction with pyroligneous solution on soil quality and wheat yield of a salt-stressed cropland from Central China Great Plain. Field Crops Res..

[36] Chen YX (2010). Effects of bamboo charcoal and bamboo vinegar on nitrogen conservation and heavy metals immobility during pig manure composting. Chemosphere.

[37] Shen R (2020). Efficient treatment of wood vinegar via microbial electrolysis cell with the anode of different pyrolysis biochars. Front. Energy Res..

[38] Liu L (2018). Effects of wood vinegar on properties and mechanism of heavy metal competitive adsorption on secondary fermentation based composts. Ecotoxicol. Environ. Saf..

[39] Fedeli R, Vannini A, Guarnieri M, Monaci F, Loppi S (2022). Bio-based solutions for agriculture: Foliar application of wood distillate alone and in combination with other plant-derived corroborants results in different effects on lettuce (*Lactuca Sativa* L.). Biology.

[40] CFIA. *Canadian Grade Compendium: Volume 2—Fresh Fruit or Vegetables*, https://inspection.canada.ca/about-cfia/acts-and-regulations/list-of-acts-and-regulations/documents-incorporated-by-reference/canadian-grade-compendium-volume-2/eng/1519996239002/1519996303947?chap=3#s34c3 (2021).

[41] Maxwell K, Johnson GN (2000). Chlorophyll fluorescence—A practical guide. J. Exp. Botany.

[42] Liu C (2019). Use of a leaf chlorophyll content index to improve the prediction of above-ground biomass and productivity. PeerJ.

[43] Fromme P, Melkozernov A, Jordan P, Krauss N (2003). Structure and function of photosystem I: Interaction with its soluble electron carriers and external antenna systems. FEBS Lett..

[44] Tripathi, D. K., Singh, V. P., Chauhan, D. K., Prasad, S. M. & Dubey, N. K. in *Improvement of crops in the era of climatic changes* (ed P. Ahmad, Wani, M., Azooz, M., Phan Tran, LS) 197–216 (Springer, 2014).

[45] Morgan SH, Lindberg S, Muhling KH (2013). Calcium supply effects on wheat cultivars differing in salt resistance with special reference to leaf cytosol ion homeostasis. Physiol. Plant..

[46] Meyer S (2006). Relationships between optically assessed polyphenols and chlorophyll contents, and leaf mass per area ratio in woody plants: A signature of the carbon–nitrogen balance within leaves?. Plant Cell Environ..

[47] Jeong C (2006). Effect of wood vinegar and charcoal on growth and quality of sweet pepper. Korean J. Hortic. Sci. Technol..

[48] Gifford RM, Evans LT (1981). Photosynthesis, carbon partitioning, and yield. Ann. Rev. Plant Physiol..

[49] Marles RJ (2017). Mineral nutrient composition of vegetables, fruits and grains: The context of reports of apparent historical declines. J. Food Compos. Anal..

[50] Liesche J, Patrick J (2017). An update on phloem transport: A simple bulk flow under complex regulation. F1000Research.

[51] Falchi R (2020). Sugar metabolism in stone fruit: Source-sink relationships and environmental and agronomical effects. Front. Plant Sci..

[52] Harris CS (2007). A single HPLC-PAD-APCI/MS method for the quantitative comparison of phenolic compounds found in leaf, stem, root and fruit extracts of *Vaccinium angustifolium*. Phytochem. Anal. Int. J. Plant Chem. Biochem. Tech..

[53] Dawson J (2017). Concentration and Content of Secondary Metabolites.

[54] Young AJ, Lowe GL (2018). Carotenoids—Antioxidant properties. Antioxidants.

[55] Ertani A (2014). *Capsicum chinensis* L. growth and nutraceutical properties are enhanced by biostimulants in a long-term period: Chemical and metabolomic approaches. Front. Plant Sci..

[56] Abou Chehade L, Al Chami Z, De Pascali SA, Cavoski I, Fanizzi FP (2018). Biostimulants from food processing by-products: Agronomic, quality and metabolic impacts on organic tomato (*Solanum lycopersicum* L.). J. Sci. Food Agric..

[57] Bhat BA (2020). Plant Micronutrients.

[58] Calvo P, Nelson L, Kloepper JW (2014). Agricultural uses of plant biostimulants. Plant Soil.

[59] Donohue, S. J., Aho, D. W. & Plank, C. O. Determination of P, K, Ca, Mg, Mn, Fe, Al, B, Cu, and Zn in plant tissue by inductively coupled plasma (ICP) emission spectroscopy. *Plant analysis reference procedures for the southern region of the United States*, 34–37 (1992).

[60] Lichtenthaler HK (1987). Chlorophylls and carotenoids: Pigments of photosynthetic biomembranes. Methods Enzymol..

[61] Dubois M, Gilles KA, Hamilton JK, Rebers PAT, Smith F (1956). Colorimetric method for determination of sugars and related substances. Anal. Chem..

[62] Ainsworth EA, Gillespie KM (2007). Estimation of total phenolic content and other oxidation substrates in plant tissues using Folin-Ciocalteu reagent. Nat. Protocols.

[63] Chang C-C, Yang M-H, Wen H-M, Chern J-C (2002). Estimation of total flavonoid content in propolis by two complementary colorimetric methods. J. Food Drug Anal..

[64] Bradford MM (1976). A rapid and sensitive method for the quantitation of microgram quantities of protein utilizing the principle of protein-dye binding. Anal. Biochem..

[65] Dudonne S, Vitrac X, Coutiere P, Woillez M, Mérillon J-M (2009). Comparative study of antioxidant properties and total phenolic content of 30 plant extracts of industrial interest using DPPH, ABTS, FRAP, SOD, and ORAC assays. J. Agric. Food Chem..

